# MST-4 and TRAF-6 expression in the peripheral blood mononuclear cells of patients with Graves’ disease and its significance

**DOI:** 10.1186/s12902-017-0161-y

**Published:** 2017-02-20

**Authors:** Ai Guo, Yan Tan, Chun Liu, Xiaoya Zheng

**Affiliations:** Department of Endocrinology, The First Affiliated Hospital of Chongqing Medical University, No.1 Youyi Street, Yuzhong District, Chongqing, 400016 China

**Keywords:** Graves’ disease, Innate immunity, TLRs

## Abstract

**Background:**

MST-4 and TRAF-6 are involved in the regulation of inflammatory and immune responses. However, whether they participate in the pathogenesis of Graves’ disease (GD) has not yet been reported. Therefore, the purpose of this study was to investigate the expression of MST-4 and TRAF-6 in the peripheral blood of patients with GD to understand their role in the pathogenesis of GD.

**Methods:**

Thirty newly diagnosed GD patients, 24 GD patients in remission (eGD) and 30 normal controls (NC) were recruited. Thyroid function and autoantibody levels were determined using a chemiluminescence immunoassay. Peripheral blood mononuclear cells (PBMCs) were extracted, and MST-4 and TRAF-6 mRNA and protein levels were determined using real-time PCR and Western blotting, respectively.

**Results:**

1. Thyroid function in the GD group was significantly different from that in the eGD and NC groups (*P <* 0.05); however, there was no difference in thyroid function between the eGD group and the NC group (*P >* 0.05). The autoantibody levels in the NC group were significantly different from those in the GD and eGD groups (*P <* 0.05); however, the difference in the levels between the GD group and eGD group was not statistically significant (*P >* 0.05). 2. The MST-4 and TRAF-6 mRNA and protein levels in the GD group were significantly lower than those in the NC group (*P <* 0.05); however, there were no differences in mRNA and protein levels between the GD group and the eGD group or between the eGD group and the NC group (*P >* 0.05). 3. The correlation between the MST-4 and TRAF-6 mRNA and protein levels was not significant. However, there was a significant correlation between the TRAF-6 mRNA and TPO Ab levels in the eGD group and between the TRAF-6 mRNA and TR Ab levels in the NC group.

**Conclusion:**

The MST-4 and TRAF-6 mRNA and protein levels were lower in the GD group than in the NC group, suggesting that MST-4 and TRAF-6 may be important in the pathogenesis of GD. Whether MST-4 influences the innate immune response through TRAF-6 and thus regulates the imbalance in downstream effector T cells requires further study. Investigating the expression of MST-4 and TRAF-6 in GD can provide a new perspective and targets for further study of the upstream mechanism responsible for effector T cell imbalance.

## Background

Graves’ disease (GD) is an organ-specific autoimmune disease that causes the level of thyroid hormone to increase. The pathogenesis of GD is still unclear; therefore, there is no effective treatment for it. The immune system plays an important role in GD, and studies have shown that imbalances in the function of effector CD4^+^ T cells (Th1, Th2, Th17 and Treg, among others) lead to the production of autoantibodies and inflammatory cytokines, which promote the disease [1–3]. However, the mechanisms underlying the imbalance in effector CD4+ T cells are unclear.

TNFR-associated factor 6 (TRAF-6), a member of the TRAF family of proteins, consists of 530 amino acids and has a molecular weight of 60 kDa. It consists of TRAF-N domains, which have a coiled-coil structure, and a conserved TRAF-C domain [4]. Because of its unique receptor-binding specificity, TRAF-6 is critical for the tumor necrosis factor receptor family (TNFR), the interleukin-1 receptor (IL-1R), the toll-like receptor (TLR) signaling pathways [5], CD40 [6] and other signaling pathways. Therefore, TRAF-6 has shown conserved function in activation of the regulation of immunity, apoptosis, stress response, inflammation and bone metabolism [7, 8], etc.

Innate immunity, an organism’s first line of defense against pathogens, is the foundation for and initiator of adaptive immunity. Toll-like receptors (TLRs), a receptor family, are the bridge connecting the innate and adaptive immune systems [9]. In the TLR signaling pathway, TRAF-6 is a central adapter molecule. When a TLR ligand binds to the TIR domain, the intracellular domain (TIR) interacts with myeloid differentiation factor 88 (MyD88). MyD88 initiates the phosphorylation of IRAK (IL-1R-associated kinase) proteins, which results in activation of the E3 ubiquitin ligase activity of TRAF-6. Subsequently, TRAF-6 catalyzes the K63-mediated ubiquitination of substrates, including TRAF-6 itself, IKKc/NEMO (NF-kB essential modulator) and the mitogen-activated protein (MAP) kinase TAK1 (TGF-β-activated kinase 1). These events are upstream of the activation of the IKKs, which comprise two kinases, IKKa and IKKb, and the catalytically inactive IKKc regulatory subunit. Together, these IKK proteins coordinate the degradation of I-kB, releasing NF-kB to translocate into the nucleus and induce the transcription of target genes [10].

The mammalian Ste20 family is a large class of serine / threonine protein kinases. The GCKs are a subfamily of the mammalian Ste20-like kinase family. The GCKs can be further subdivided into GCK-I to GCK-VIII [11]. Mammalian Ste20-like kinase 4 (MST-4) is a member of the GCKIII subfamily. MST-4 consists of 416 amino acids and has a molecular weight of 46 kDa. The gene is located on chromosome Xq26. MST-4 consists of a C-terminal regulatory domain and an N-terminal kinase domain [12]. It is widely expressed at various levels in many tissues, such as high expression in the placenta, moderate expression in the brain, heart, lungs, liver, muscle, kidney, and pancreas and low expression in skeletal muscle. MST-4 plays a role in promoting the growth of cells, cell polarization and orientation of the Golgi apparatus.

The latest research suggests that MST-4 has an important regulatory function in innate immunity. In the TLR signaling pathway, MST-4 directly associates with and phosphorylates TRAF-6, impairing its oligomerization and ubiquitination activity, which can lead to the abnormal function of downstream signaling molecules. As a result, MST-4 inhibits the activation of inflammatory pathways, regulating the production of downstream inflammatory mediators [13]. Thus, the abnormal expression of MST-4 may be involved in autoimmune diseases. This study provides a new perspective for the study of immune- and inflammatory-related diseases.

In summary, MST-4 and TRAF-6 are involved in the regulation of inflammatory and immune responses. However, whether they participate in the pathogenesis of GD has not yet been reported. Therefore, the purpose of this study was to investigate the expression of MST-4 and TRAF-6 in the peripheral blood of patients with GD, to understand their role in the pathogenesis of GD, and to provide a new understanding of the mechanism regulating GD.

## Methods

### Study subjects

Patients with GD and healthy persons were recruited from the First Affiliated Hospital of Chongqing Medical University from September 2015 - April 2016. The subjects were divided into a GD group, a GD remission (eGD) group and a normal control (NC) group. According to the guidelines for the diagnosis and management of thyroid disease presented by the American Thyroid Association(ATA) and the American Association of Clinical Endocrinologists (AACE) in 2011 [14], the GD group inclusion criteria were as follows: (1) symptoms of elevated metabolism; (2) increased thyroid hormone concentration, and decreased serum TSH concentration; (3) diffuse thyroid enlargement (palpation and B-confirmed) with or without goiter; (4) anterior tibial mucinous edema; (5) eye bulging and other infiltrative ophthalmopathy; (6) positive for TR Ab, TS Ab, TPO Ab, and Tg Ab. For the above criteria, (1)-(3) are a prerequisite for the diagnosis of GD, (4)-(6) are the diagnosis of auxiliary conditions. The GD group included newly diagnosed untreated patients, a total of 30 cases, including 19 females and 11 males. The inclusion criteria for the eGD group were as follows: (1) GD was diagnosed; (2) anti-thyroid drug (methimazole) treatment for ≥ 1 year; (3) a normal level of thyroid function with a maintenance dose of 2.5–10 mg / d. The eGD group included a total of 24 cases, including 18 females and 6 males. The NC group was consisted of 30 healthy age- and sex-matched volunteers as normal controls, including 21 females and 9 males. The exclusion criteria included acute and chronic infections, other autoimmune diseases (systemic lupus erythematosus, rheumatoid arthritis, psoriasis, etc.), tumors, pregnancy, and the recent consumption of iodine-containing foods or drugs.

The study was approved by the First Affiliated Hospital of Chongqing Medical University Ethical Committee. All participants were provided informed consent when being enrolled and fully explained the purpose and procedures of the study. Informed consent was obtained from all participants.

### Experimental materials

The following reagents were used in this study: human lymphocyte separation medium (TBD Tianjin Biotech, China); RNAiso Plus, Reverse Transcription and Amplification kit (TaKaRa, Japan); cell lysis buffer for Western, BCA Protein Assay Kit, SDS-PAGE gel preparation kit, Prestained Color Protein Molecular Weight Marker (Beyotime, China); WesternBright ECL reagent (Advansta, USA); rabbit anti-human TRAF-6 and anti-MST-4 antibodies (CST, USA); rabbit anti-human β-actin antibody (Proteintech, China); horseradish peroxidase (HRP)-labeled goat anti-rabbit IgG secondary antibody (Proteintech, China); nonfat dry milk (Bio-Rad, USA); PVDF membrane (Millipore, USA); and thyroid function and autoantibody kits and detection equipment (Beckman Coulter, Inc., USA).

### Specimen collection

Twelve milliliters of peripheral blood were collected in EDTA anticoagulation Vacutainers; 2 ml were used for detecting antibodies and thyroid function indicators, while the other 10 ml were used for peripheral blood mononuclear cell (PBMC) extraction.

### Detection of thyroid function indicators

The Beckman Coulter UniCel DxI 800 automatic immunoassay analyzer and reagents were used to evaluate thyroid function (FT3, FT4, and TSH) and antibody levels (TRAb, TGAb, and TPOAb). Detection was completed by the hospital endocrine laboratory technicians. The normal reference values for the thyroid function indicators and antibodies used in our hospital are as follows: FT3: 2.5-3.9 pg/ml; FT4: 0.61-1.12 ng/dl; TSH: 0.35-3.5 IU/ml; TG Ab: < 4 IU/ml; TPO Ab: < 9 IU/ml; and TR Ab: 0.3-1.8 IU/L.

### Extraction of PBMCs

Ficoll density gradient centrifugation was used to isolate PBMCs. One-third of the total PBMCs (approximately 3–4 × 10^6^) were used for the RNA extraction, whereas the remaining 2/3 (approximately 6–8 × 10^6^) were used for the total protein extraction.

### Total RNA extraction and the determination of MST-4 and TRAF-6 mRNA expression via RT-PCR

The expression of MST-4 and TRAF-6 was measured using RT-PCR. Total RNA was extracted from PBMCs. cDNA was synthesized using an RT Reagent Kit (TaKaRa, Japan). The primer sequences are shown in Table 1. The following program was run for 40 cycles: 95 °C, 30 s; 95 °C, 5 s; 60 °C, 30 s. The reactions contained the following reagent proportions: 5 μl SYBR® Premix Ex Taq II (Tli RNaseH Plus) (2 ×), 1 μl upstream primer, 1 μl downstream primer, 1 μl cDNA, and 2 μl RNase-free dH_2_O. The threshold cycle (cycle threshold, Ct) was obtained with a fluorescence quantitative PCR instrument (Bio-Rad, USA). The relative levels of MST-4 and TRAF-6 mRNA were calculated using the 2-ΔΔCt method and were normalized to the corresponding β-actin values.

**Table 1:** Primer sequences used for the real-time PCR

Primer	Sequences	bp
MST-4	F 5′ TGAGGAAGCCGAAGATGAAATAG 3′ R 5′ CCAGCTCGAAGAAGATCCAGTG 3′	170
TRAF-6	F 5′ GGATTCTACACTGGCAAACCCG 3′ R 5′ CCAAGGGAGGTGGCTGTCATA 3′	137
β-actin	F 5′ CCACGAAACTACCTTCAACTCC 3′ R 5′ GTGATCTCCTTCTGCATCCTGT 3′	132

### The expression of MST-4 and TRAF-6 protein

Total cellular protein was extracted from PBMCs using the Reagent kit (Beyotime, China). The protein concentration was measured with a Protein Assay Kit (Beyotime, China). Equal amounts of protein per sample were separated by SDS–PAGE and transferred to a PVDF membrane. The membrane was blocked with 5% nonfat milk in Tris-buffered saline with 0.05% Tween20 (TBST) for 3 h. The membranes were washed 3 times for 10 min each in TBST and incubated with monoclonal rabbit anti-human TRAF-6 (1:1000), monoclonal rabbit anti-human MST-4 (1:1000), and monoclonal rabbit anti-human β-actin antibody (1: 2000), followed by horseradish peroxidase (HRP)-labeled goat anti-rabbit IgG secondary antibody (1:5000). Immunoreactive bands were developed using the WesternBright ECL reagent (Advansta, USA). For the image analysis, the films were scanned and analyzed using a chemiluminescence system (VILBER FUSION FX5, France).

### Data analysis

Statistical analysis was performed with SPSS software (IBM, Armonk, NY, version 19.0). The results were expressed as x ± s. One-way analysis of variance combined with the Bonferroni test was used for statistical analysis of the data. Correlation between variables was determined with the Pearson correlation coefficient; *P-*values less than 0.05 were considered statistically significant.

## Results

### Thyroid function and autoantibody levels

The levels of the indicators of thyroid function (FT3 and FT4) were higher, whereas TSH was lower in the NC group than in the eGD group, and these differences were statistically significant (*P <* 0.001); however, the differences between the eGD group and the NC group were not statistically significant (*P >* 0.05). The level of autoantibodies (TR Ab, TG Ab, and TPO Ab) differed significantly between the GD group and the NC group and between the eGD group and the NC group (*P =* 0.000), but the difference between the eGD group and NC group was not statistically significant (*P >* 0.05), as shown in Table 2.

**Table 2:** The levels of indicators of thyroid function and autoantibodies among the three groups $\left(\overline{x}\pm s\right)$

	GD group	eGD group	NC group
Age (years)	39.00 ± 19.22	37.13 ± 1 1.78	39.97 ± 13.73
FT3 (pg/ml)	10.87 ± 7.85^①②^	3.19 ± 0.28	3.14 ± 0.37
FT4 (ng/dl)	3.28 ± 1.71 ^①②^	0.90 ± 0.13	0.78 ± 0.20
uTSH (μIU/ml)	0.03 ± 0.02^①②^	1.57 ± 0.73	1.90 ± 0.83
TRAb (ng/ml)	11.30 ± 8.80^①^	7.90 ± 2.64^①^	0.64 ± 0.34
TPOAb (IU/ml)	109.50 ± 108.04^①^	104.84 ± 143.37^①^	1.16 ± 1.10
TGAb(IU/L)	56.95 ± 56.15^①^	45.77 ± 76.53^①^	0.2 ± 0.14
Total (F/M)	30 (19/11)	24 (18/6)	30 (21/9)

^①^ *P <* 0.05 compared with the normal group; ^②^ *P <* 0.05 compared with the eGD group

### The expression of MST-4 and TRAF-6 mRNA in the PBMCs of each group

**Fig. 1:**
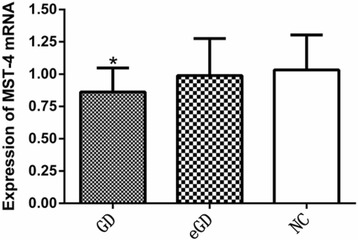
Expression of MST-4 mRNA in PBMCs *Compared with the normal group

**Fig. 2:**
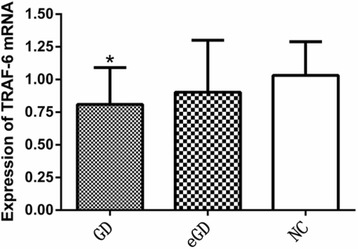
Expression of TRAF-6 mRNA in PBMCs *Compared with the normal group

The expression of MST-4 and TRAF-6 mRNA in the GD group was lower than that in the NC group; these differences were statistically significant (*P <* 0.05, *P =* 0.024, *P =* 0.019); However, the differences were not statistically significant between the GD group and the eGD group or between the eGD group and the NC group (*P >* 0.05), as shown in Table 3, Figs. 1 and 2.

**Table 3:** Expression of MST-4 and TRAF-6 mRNA $\left(\overline{x}\pm s\right)$

	GD group	eGD group	NC group
MST-4	0.86 ± 0.19^①^	0.99 ± 0.29	1.03 ± 0.27
TRAF-6	0.81 ± 0.28^①^	0.90 ± 0.40	1.03 ± 0.26

^①^ *P <* 0.05 compared with the normal group

### The levels of MST-4 and TRAF-6 protein in the PBMCs of each group

**Fig. 3:**
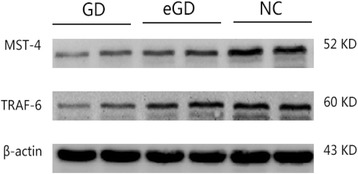
Expression of MST-4 and TRAF-6 protein, as detected using Western blotting

**Fig. 4:**
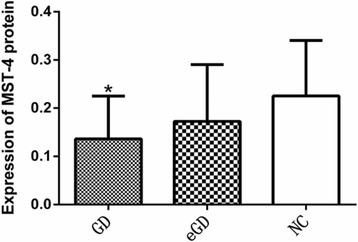
Expression of MST-4 protein in PBMCs *Compared with the normal group

**Fig. 5:**
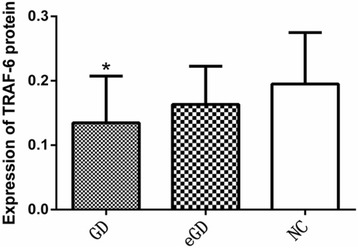
Expression of TRAF-6 protein in PBMCs *Compared with the normal group

The levels of MST-4 and TRAF-6 protein were lower in the GD group than those in the NC group, and these differences were statistically significant (*P <* 0.05, *P =* 0.0051, *P =* 0.0047). However, the differences were not statistically significant between the GD group and the eGD group or between the eGD group and the NC group (*P >* 0.05), as shown in Table 4, Figs. 3, 4, and 5.

**Table 4:** Expression of MST-4 and TRAF-6 protein $\left(\overline{x}\pm s\right)$

	GD group	eGD group	NC group
MST-4	0.14 ± 0.09^①^	0.17 ± 0.12	0.23 ± 0.12
TRAF-6	0.13 ± 0.07^①^	0.16 ± 0.06	0.20 ± 0.08

^①^ *P <* 0.05 compared with the normal group

### Correlation analysis

The expression of MST-4 mRNA was not significantly correlated with the level of TRAF-6 mRNA, thyroid function, or the level of autoantibodies in any of the groups. However, the expression of TRAF-6 mRNA was positively correlated with thyroid function and the level of TPO Ab in the eGD group (*r =* 0.4291, *P =* 0.0364) and negatively correlated with the level of TR Ab in the NC group (*r =* −0.4085, *P =* 0.025). There was no significant correlation between the MST-4 or TRAF-6 protein levels and thyroid function or autoantibody levels in any group (*P >* 0.05).

## Discussion

MST-4 has been found to participate in a variety of biological functions in cells since it was identified in 2001. Studies have shown that MST-4 promotes cell proliferation to influence the development of neoplastic disease [15–17]. In addition, MST-4 can activate the LKB 1-STRAD-MO25 complex, which is involved in cell polarization [18]. MST-4 can also interact with cerebral cavernous malformations (CCM3) to participate in cell migration and orientation of the Golgi [19]. However, whether MST-4 regulates the immune response was unclear. The other members of the GCK family play important roles in the innate and adaptive immune responses, but each has a different regulatory mechanism. The GCK-1 family member, MAP4K2, is involved in pathogen-associated molecular pattern (PAMP) signaling pathways and plays a role in the JNK and p38 pathways [20]. The GCK-II member MST1 is involved in negative regulation of T cell proliferation [21] and plays a key role in lymphocyte chemotaxis and thymocyte emigration [22, 23]. The GCK-VI member Ste20-like proline / alanine-rich kinase (SPAK) is involved in the TCR / CD28-induced activation of the transcription factor AP-1 [24]. The GCK-VII member TAO2 can activate the p38 signaling pathway to regulate the expression of inflammatory cytokines via extracellular signal-regulated kinase kinase 3 (MEK3) and MEK6 [25].

There are still few recent studies about the role of GCK-III family members in the immune mechanism. Therefore, the Chinese group Zhou et al. [21] was the first to explore the role of the MST-4 in the innate immune response in 2015. An analysis of clinical samples from patients with sepsis caused by infection revealed decreased MST-4 levels. In vivo and in vitro experiments revealed that the expression of MST-4 responded dynamically to LPS stimulation and that the phosphorylation of TRAF-6 affected its activity, which caused the signaling information not to be delivered and suppressed the production of the pro-inflammatory cytokines IL-6 and TNF-α. Therefore, MST-4 can participate in the regulation of TLR signaling pathways. Furthermore, mouse models of septic shock were used to further investigate MST-4 function. It was found that MST-4 knockdown could cause a more severe inflammatory response. The study revealed that MST-4 played a similar role in inhibiting an excessive immune response to protect the body. Thus, MST-4 provided a new perspective for the study of immune and inflammatory-related diseases [13].

The imbalance in effector T cells at the level of adaptive immunity plays an important role in GD, but the pathogenesis of innate immunity in GD is poorly understood. Studies have found that polymorphisms in and abnormal expression of TLR receptor genes [26–28] and alterations in the functional activity of antigen-presenting cells (DCs) are important in GD [29, 30] and in other autoimmune diseases, which suggested the functional activation of immune cells (DCs, monocytes, macrophages, etc.). The activation of the TLR signaling pathway influences the proliferation and differentiation of downstream CD4+ effector T cells, augmenting the immune and inflammatory responses and leading to the onset of GD. However, the innate immune mechanism underlying GD still is not entirely clear, and it is necessary to further explore the mechanism of the disease and find a better explanation for the adaptive immune imbalance. Therefore, we need a better understanding of the molecules and related mechanisms involved in regulating the TLR signaling pathway in innate immunity, and we must study the regulatory mechanisms underlying the imbalance in effector CD4+ T cells. As a novel target, MST-4 provides an opportunity to further our understanding of the role of innate immunity in the pathogenesis of GD.

In this study, we first found that the expression of MST-4 mRNA and protein in the GD group was lower than that in the NC group, which was consistent with the results observed in sepsis due to infection [13]. This result indicated that the expression of MST-4 is abnormal in GD. However, the expression of MST-4 was not significantly different between the GD group and the eGD group, or between the eGD group and the NC group, suggesting that the immune inflammatory statue in the eGD group is probably between that of the GD group and the normal controls, which contributed to the above results. However, further study is required to determine whether MST-4 aberrantly regulates TRAF-6 in TLR signaling pathways, promotes the aberrant activation of TLR signaling pathways, activates antigen-presenting cells (APCs), and stimulates downstream effector T cell proliferation and differentiation, further promoting the onset of GD. The present study was the first to explore the expression of MST-4, decreases in which may lead to abnormal innate immune responses, in the autoimmune disease GD. Therefore, a foundation for further study of the regulatory mechanisms of MST-4 in the innate immune response was provided by this study in GD.

TRAF-6 is an important adapter molecule in the innate and adaptive immune responses. Aberrant expression of TRAF-6 may cause the aberrant activation of signaling pathways, resulting in activation of downstream inflammatory responses and the development of immune-related inflammatory diseases. Therefore, it was unclear whether the aberrant expression of TRAF-6 in GD is involved in immune and inflammatory responses and regulates the pathogenesis of GD. This study found that the levels of TRAF-6 mRNA and protein were lower than normal, suggesting that TRAF-6 may have a role in the pathogenesis of GD. However, there were no significant differences in the expression of TRAF-6 between the GD group and the eGD group or between the eGD group and the NC group, suggesting that the immune inflammatory status in the eGD group was remission after treatment, but it did not return to the normal level.

In this study, the decrease in TRAF-6 was consistent with other findings. For example, the expression of TRAF-6 in patients with untreated ankylosing spondylitis (AS) was significantly lower than that in a control group, suggesting that the abnormal expression of TRAF-6 may play a role in the pathogenesis of AS [31]. However, the expression of TRAF-6 in some other autoimmune diseases exhibits the opposite trend. For example, the TRAF-6 level in the synovial tissue of patients with RA was higher than that in the synovial tissue of patients with osteoarthritis (OA), suggesting that TRAF-6 mediates the inflammatory response involved in synovial inflammation and joint destruction in RA [32]. A study demonstrated that two members of the TRAF family, TRAF4 and TRAF-6, were activated in patients with inflammatory bowel disease (IBD) and that both TRAF4 and TRAF-6 showed potential diagnostic value in differential diagnosis [33]. The protein levels of the TLRs, TRAF-6, MyD88, and NF-κB were significantly increased in the adipose tissue of patients with type 2 diabetes [34].

The down-regulation of TRAF-6 in GD may be associated with the presence of other proteins inhibiting TRAF-6 signal transduction. For example, heat shock protein 70 (HSP70) can associate with the C-terminal TRAF domain, prevent its ubiquitination, and inhibit NF-κB activation [35]. Suppression of cytokine signaling-3 (SOCS-3) inhibited TRAF-6 ubiquitination to prevent TRAF-6 and TAK1 interactions [36]. Multifunctional proteins in the β-arrestin family can form a complex with TRAF-6 to prevent TRAF-6 ubiquitination and signal transduction. A zinc finger-like protein (ZCCHCll) also inhibited TRAF-6 signaling [37]. Thus, the decreased expression of TRAF-6 in GD might affect both the regulatory role of MST-4 and the combined effects of various effector protein molecules. The expression of TRAF-6 may be associated with the dynamics of the disease: when the immune system is activated, both the inflammatory response and the related inhibitory factors increase, which requires balancing of the immune and inflammatory responses in order for the body to avoid excessive induction of an immune response.

Studies have shown that TRAF-6 is also essential in immune tolerance. Previous studies have also demonstrated that knocking out TRAF-6 in mouse T cells can cause inflammation in multiple organs and mononuclear cell infiltration into the intestine, liver, lung, and kidney. Higher levels of IL-4 and IL-5 are produced by TRAF-6-deficient T cells, and these mice develop an inflammatory disease mediated by activated Th2 cells. In addition, the serum IgG1, IgE, IgM and anti-DNA autoantibody levels were significantly elevated in TRAF-6-specific T cell-deficient mice, resulting in an enhanced humoral immune response. Therefore, TRAF-6-deficient T cells can affect immune homeostasis, and autoimmune disease can appear [38]. Studies have further found that Treg-specific TRAF-6 knockout (CKO) mice may develop allergic skin diseases, arthritis, swollen lymph nodes and a hyper immunoglobulin E phenotype. Although TRAF-6-knockout Tregs had similar inhibitory activity to wild-type Tregs in vitro, the reduced number of Foxp3-positive cells suggests that TRAF-6 knockout cannot suppress the development of colitis in mice with lymphopenia. These data suggest that TRAF-6 plays an important role in the regulation of T cells, primarily by maintaining Foxp3 regulatory T cells and inhibiting pathogenic Th2-type conversion [39]. In addition, TRAF-6 deficiency may lead to immune tolerance imbalance. Considering that GD is an autoimmune disease, there may be an imbalance in immune tolerance. Thus, in GD, a decreased level of TRAF-6 may also be associated with an imbalance of immune tolerance. However, further study is required to determine whether TRAF-6 participates in the abnormal regulation of Treg cells and promotes the development of GD.

Phosphorylated TRAF-6 (mediated by MST-4) primarily takes part in the immune response. Therefore, total protein and phosphorylated TRAF-6 levels should be detected. Typically, phosphorylation-specific antibodies cannot detect total protein levels, and total protein antibodies can detect all forms of the protein, including the phosphorylated and non-phosphorylated forms. This study examined the expression of total TRAF-6 protein, but not phosphorylated TRAF-6, mainly because phospho-TRAF-6-specific antibodies suitable for Western blot are not currently available commercially. Thus, we did not evaluate phosphorylated TRAF-6. A co-immunoprecipitation assay can measure phosphorylated TRAF-6, but due to the limited amount of peripheral blood samples, we failed to detect phosphorylation of TRAF-6. In this study, the level of total TRAF-6 protein was decreased, suggesting that the phosphorylation of TRAF-6 may also be lower. Although there was no correlation between MST-4 and TRAF-6 levels, this did not completely eliminate the possibility that MST-4 might play a regulatory role in the phosphorylation of TRAF-6 and that the reduction in TRAF-6 levels occurred due to the influence of other regulatory molecules. Therefore, we must study the relationship between MST-4 and phosphorylated TRAF-6 further and analyze whether altered MST-4 expression has a direct effect on TRAF-6 phosphorylation.

This study has some shortcomings, such as the relatively small number of samples collected for each group; thus, the role of MST-4 and TRAF-6 in the pathogenesis of GD must be studied in a large sample to verify these data. It is well known that GD is an organ-specific autoimmune disease. Therefore, it is important to comprehensively evaluate the influence of MST-4 and TRAF-6 in the thyroid gland to fully understand the effects of MST-4 and TRAF-6 in GD. The PBMCs in this study consisted of various types of cells, including lymphocytes (approximately 70 to 90% of PBMCs), monocytes, (approximately 10 to 30%), and dendritic cells (approximately 1 to 2%). Therefore, the expression level determined may be the combined effect of a number of factors. Studying the role of MST-4 in the regulation of TRAF-6 requires the investigation of a relatively specific cell population to eliminate confounding factors.

## Conclusion

This study showed that the expression of MST-4 and TRAF-6 was decreased in GD, suggesting the involvement of these molecules in the pathogenesis of GD. However, whether MST-4 had an effect on the interaction between the innate immune response and TRAF-6 in GD and the mechanism responsible for the imbalance in downstream effector T cells require further study. The present study explored the expression of MST-4 and TRAF-6 in GD and provided a new perspective and targets for further study of the upstream mechanism underlying the effector T cell imbalance, which plays a key role in GD pathogenesis. This research provides a new direction for studying the pathogenesis of GD and a new target for the effective diagnosis and treatment of GD pathogenesis.

## Abbreviations

AACE: The American Association of Clinical Endocrinologists
ATA: The American Thyroid Association
FT3: Free triiodothyronine
FT4: Free thyroxine
GD: Graves’ disease
MST-4: Mammalian Ste20-like kinase 4
Tg Ab: Thyroglobulin antibody
TLR: Toll-like receptor
TPO Ab: Thyroid peroxidase antibody
TR Ab: Thyrotropin receptor antibody
TRAF-6: TNFR-associated factor 6
uTSH: Ultra-sensitive thyrotropin
